# Protocols for Co-Culture Phenotypic Assays with Breast Cancer Cells and THP-1-Derived Macrophages

**DOI:** 10.1007/s10911-024-09556-2

**Published:** 2024-02-10

**Authors:** Alicja Mazan, Anna A. Marusiak

**Affiliations:** Laboratory of Molecular OncoSignalling, IMol Polish Academy of Sciences, Flisa 6, Warsaw, 02-247 Poland

**Keywords:** Co-culture, Macrophages, THP-1, Tumor microenvironment, Breast cancer

## Abstract

Tumor mass comprises not only cancer cells but also heterogeneous populations of immune and stromal cells, along with the components of the extracellular matrix, collectively called the tumor microenvironment (TME). This diverse population of cells can communicate with each other, which can positively or negatively affect tumor growth and progression to malignancy. The most common type of immune cells in the TME are macrophages. Macrophages continuously differentiate into a broad landscape of tumor-associated macrophages (TAMs) in response to numerous signals from the TME, which makes studies on TAMs quite challenging. Therefore, implementing reliable protocols is a milestone for drawing consistent conclusions about the interactions between cancer cells and TAMs. Here, we provide the details for the polarization of a human leukemia monocytic cell line, THP-1, into M0, M1 and M2 macrophages. We also present a step-by-step protocol for a transwell co-culture using a human breast cancer cell line, HCC1806, and THP-1-derived macrophages. Finally, we describe the colony formation and migration assays performed on the breast cancer cells after the co-culture with macrophages to measure the influence of macrophages on the oncogenic features of cancer cells. In summary, our co-culture-based protocols can be a valuable resource for investigating the interactions between macrophages and cancer cells.

## Introduction

It has become evident in recent years that cancer cells do not act alone but closely cooperate with their surrounding environment [1]. The composition of the tumor microenvironment (TME) is very variable and consists of non-cancer cells (including immune cells, hematopoietic cells, adipocytes and fibroblasts), soluble signal factors secreted by these cells and extracellular matrix [2]. The TME plays a crucial role in initiating oncogenesis, disease progression, metastasis and response to anticancer therapies. The interactions between cancer cells and the components of the tumor microenvironment are dynamic and depend on the stage of cancer development and the treatment. They are also crucial in inducing both pro-tumorigenic and anti-tumorigenic events.

Immune cells play an essential role in the development of cancer. Tumor-associated macrophages (TAMs) are the most common type of immune cells found in the TME, and they can comprise up to 50% of the solid tumor mass [3]. High heterogeneity and plasticity are typical features of macrophages as they undergo dynamic changes in their response to the microenvironment. Depending on the stimulation factors, macrophages can be polarized into two phenotypes: the classically activated M1 and the alternatively activated M2 [4]. M1 and M2 macrophages have distinct functions in the organism, and the balance between them is crucial for homeostasis and inflammatory responses. While M1 macrophages are known to promote inflammatory responses, M2 macrophages are generally associated with tumor-promoting conditions in the TME. A high percentage of TAMs in the TME correlates with poor survival of cancer patients [5, 6]. Several studies have shown complex bidirectional dependencies between cancer cells and TAMs [7]. For example, the interaction between TAMs and malignant cells results in enhanced cancer cell proliferation, the transition towards mesenchymal phenotype, promotion of angiogenesis and elevated expression of cytokines [8–12]. Moreover, TAMs at the invasive front of tumors lead to increased invasion and metastasis of cancer cells [8, 11]. Macrophages are also essential in establishing an immunosuppressive environment that aids tumors in evading immune responses [13]. On the other hand, cancer cells can also alter the tumor microenvironment by the polarization of macrophages towards the M2 phenotype [13–17]. These examples highlight the importance of a feedback loop between TAMs and cancer cells in the progression towards malignant phenotype. Uncovering the molecular basis of these interactions has become a broad interest as it opens up possibilities for developing novel anti-cancer therapies.

Currently, co-culture techniques that allow the study of tumor microenvironment interactions are in the developmental phase. However, co-culture methods vary between laboratories, leading to inconsistent data interpretation. Additionally, the scientific literature lacks detailed protocols for co-culture techniques, which hinders obtaining reproducible results between studies and limits the ability to repeat the procedures. Here, we describe the methods used within our laboratory to perform phenotypic assays based on the transwell co-culture of breast cancer cells and macrophages. We use THP-1, a human leukemia monocytic cell line that can be differentiated into M0, M1 and M2 macrophages [18]. We determined the optimal conditions for macrophage polarization into M0, M1 and M2 subtypes. The expression of M1 and M2 markers was confirmed via two independent techniques: flow cytometry and RT-qPCR. Finally, we provided the protocols for conducting colony formation and migration assays for breast cancer cells, HCC1806, after co-culturing them with THP-1-derived macrophages. Thanks to these methods, we can effectively examine the impact of macrophages on the clonogenic potential and migratory activity of breast cancer cells.

## Reagents, Solutions and Materials

1. Culture medium: 10% of fetal bovine serum (FBS; F7524-500ML, Sigma-Aldrich), 100 U/mL penicillin, 100 µg/mL streptomycin (P4333-100 ml, Sigma-Aldrich), 1 mM sodium pyruvate (S8636-100ML, Sigma-Aldrich) in RPMI-1640 medium (21875034, Gibco).
2. THP-1 cell line (ACC 16, DSMZ) (see *Note 1*).
3. HCC1806 cell line (a kind gift from CRUK Manchester Institute UK; CRL-2335, ATCC).
4. Trypsin (T4049-100ML, Sigma-Aldrich).
5. Phosphate-buffered saline (PBS; 10010023, Gibco).
6. Bovine serum albumin (BSA; PAO20QF065, Biowest).
7. FACS buffer - PBS with 1% BSA.
8. Human TruStain FcX (Fc receptor blocking solution) (422302, BioLegend).
9. BD FACS Sheath Fluid (342003, BD Biosciences).
10. BV421 Mouse Anti-Human CD206 (564062, BD Biosciences).
11. PE Mouse Anti-Human CD38 (555460, BD Biosciences).
12. Dimethyl sulfoxide (DMSO; A994.1, ROTH).
13. Phorbol 12-myristate 13-acetate (PMA; 79346-5MG, Sigma-Aldrich). Reconstitute PMA powder by adding DMSO (sterile, cell culture grade). Next, prepare a working solution in water (sterile, cell culture grade). Aliquot and store at − 80 °C (see *Note 2*).
14. IL13 (I1771-10UG, Sigma-Aldrich). Reconstitute IL13 in sterile PBS containing 0.1% BSA. Aliquot and store at − 80 °C.
15. IL4 (H7291-10UG, Sigma-Aldrich). Reconstitute IL4 in sterile PBS containing 0.1% BSA. Aliquot and store at − 80 °C.
16. IFNgamma (SRP3058-100UG, Sigma-Aldrich). Reconstitute IFNgamma in sterile PBS containing 0.1% BSA. Aliquot and store at − 80 °C.
17. Lipopolysaccharide (LPS; SMB00610, Sigma-Aldrich). Reconstitute LPS in sterile PBS containing 0.1% BSA. Aliquot and store at − 80 °C.
18. Methanol (179957-1 L, Sigma-Aldrich).
19. Crystal Violet (C0775-25G, Sigma-Aldrich). Prepare 0.5% crystal violet solution in 25% methanol. Mix well and store at room temperature.
20. Shandon Formal-Fixx™ Concentrate (6764254, Thermo Fisher Scientific). Prepare 1x solution by mixing 1 part of the Formal-Fixx™ Concentrate with 4 parts of miliQ water.
21. GeneMATRIX Universal RNA Purification Kit (E3598, EurX).
22. iTaq Universal SYBR^®^ Green Supermix (1725121, Bio-Rad).
23. High-Capacity cDNA Reverse Transcription Kit (4368814, Applied Biosystems™).
24. Transwell^®^ polycarbonate membrane cell culture inserts, 0.4 μm (3412, Corning) (see *Note 3*).
25. Permeable support for 12-well plate with 8.0 μm transparent PET membrane (353182, Corning) (see *Note 3*).
26. Costar^®^ 12-well clear TC-treated multiple well plates, individually wrapped, sterile (3513, Corning).
27. Costar^®^ 6-well clear TC-treated multiple well plates, individually wrapped, sterile (3516, Corning).
28. 96-well PCR plates (402712, Nest Scientific Biotechnology).
29. Polystyrene assay plates, 96-Well, U-bottom (732–3720, VWR).
30. Round-bottom polystyrene test tubes with cell strainer snap cap, 5mL (352235, Corning).
31. Falcon^®^ cell culture flasks (353136, Corning).
32. Nunc™ flasks, non-treated (156800, Thermo Fisher Scientific).

## Methods

### Generation of M0, M1 and M2 Macrophages

**Fig. 1:**
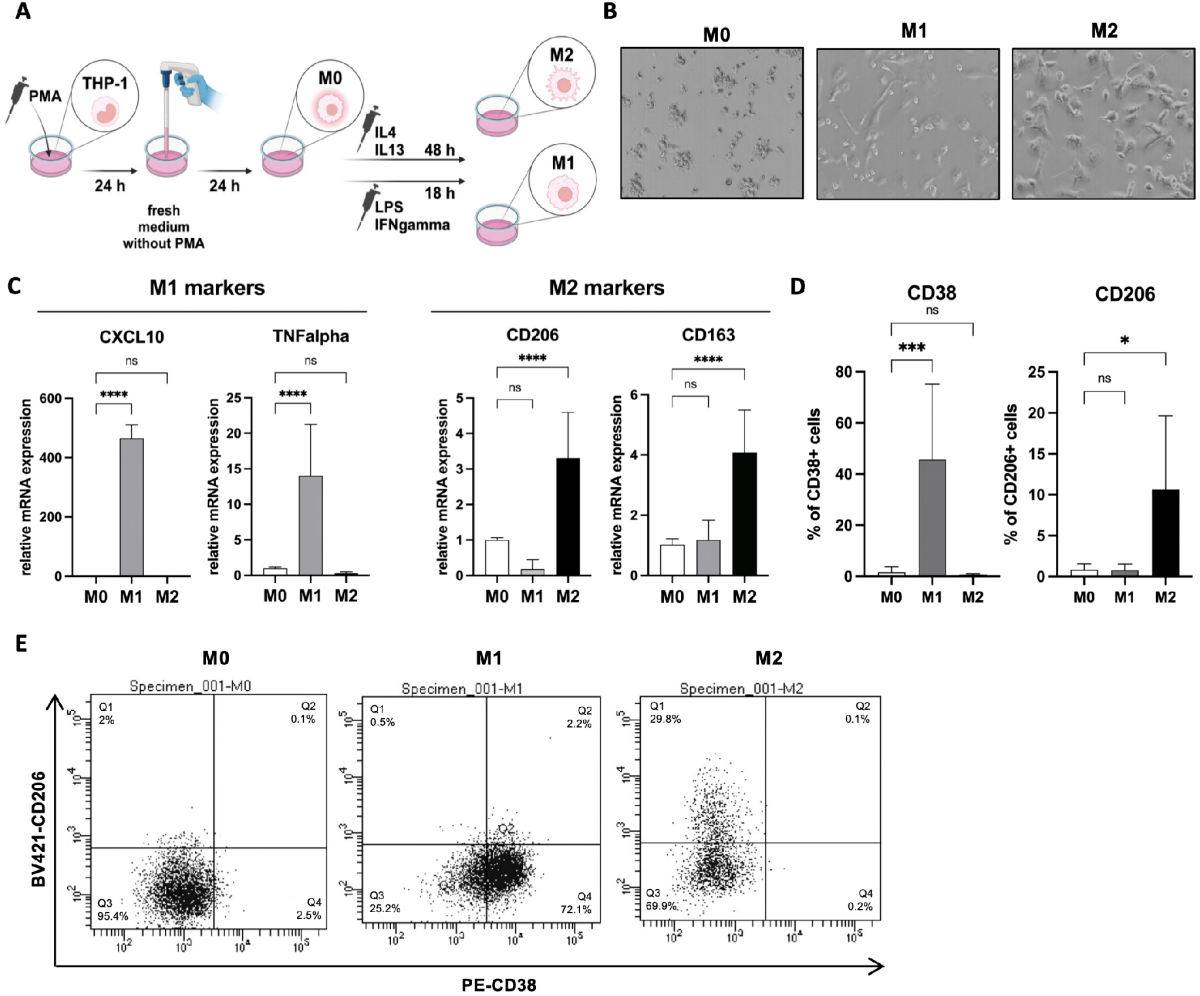
M0, M1 and M2 macrophage generation and validation by RT-qPCR and flow cytometry. **A** Schematic illustration of M0, M1 and M2 generation. **B** Representative pictures of THP-1-derived M0, M1 and M2 macrophages. **C** THP-1 were treated with 150 nM PMA for 24 h, followed by a 24 h rest period with fresh culture media. For M1, cells were incubated with 100 ng/mL LPS, and 20 ng/mL IFNgamma for 18 h. For M2, cells were incubated with 20 ng/mL IL4, and 20 ng/mL IL13 for 48 h. Following macrophage generation, RNA was isolated, and relative gene expression of M1 markers (CXCL10 and TNFalpha) and M2 markers (CD206 and CD163) was analyzed by RT-qPCR. Data represent mean results from three independent experiments (error bars ± SD). Significance was calculated using one-way ANOVA followed by the Tukey multiple comparisons test, ****p < 0.0001. **D, E** Following macrophage generation, cells were stained with M1 marker PE-CD38 and M2 marker BV421-CD206, and analyzed by flow cytometry. **D** Data represent mean results from three or more independent experiments (error bars ± SD). Significance was calculated using one-way ANOVA followed by the Tukey multiple comparisons test, *p < 0.05, ***p < 0.001. **E** Representative dot plots from flow cytometry analysis of M0, M1 and M2 macrophages stained with PE-CD38 and BV421-CD206

Depending on the experimental design, macrophages can be generated using either 6-well cell culture plates or 0.4 μm pore size transwell inserts (for co-culture experiments). The following protocol describes the 6-well plate approach (Fig. 1A). For co-culture assays described below, the same protocol should be followed, but the THP-1 cells should be seeded in 0.4 μm pore size transwell inserts suitable for 6-well plates.

1. **(Day 0)** Centrifuge THP-1 cells at 190 x g for 5 min at room temperature.
2. Discard supernatant.
3. Resuspend the cell pellet in 10 mL of fresh culture medium.
4. Determine the concentration of THP-1 cells using a cell counter and staining with trypan blue.
5. Prepare THP-1 cell line suspension at 2 × 10^5^ cells per 1 mL.
6. Mix THP-1 cells suspension again and seed 2 mL to each well of the 6-well plate (4 × 10^5^ cells per well).
7. Add PMA to a final concentration of 150 nM (see *Note 2*).
8. Mix gently by swaying the 6-well plate on the horizontal and vertical axis.
9. Culture the macrophages for 24 h in the incubator at 37 °C, 5% CO_2_.
10. **(Day 1)** Check if THP-1 cells have become adherent to determine the proper polarization of M0 macrophages (Fig. 1B).
11. Aspirate off medium from wells using a glass Pasteur pipette.
12. Wash M0 macrophages with PBS.
13. Add 2 mL of fresh culture medium to each well and culture the macrophages for 24 h in the incubator at 37 °C, 5% CO_2_.
14. **(Day 2)** Differentiate the rested macrophages (M0) using a mix of cytokines:
  - Add lipopolysaccharide (LPS) to a final concentration of 100 ng/mL and interferon gamma (IFNgamma) to a final concentration of 20 ng/mL to differentiate M0 macrophages to classically-activated M1 macrophages. Culture the macrophages for 18 h in the incubator at 37 °C, 5% CO_2_.
  - Add interleukin 4 (IL4) and interleukin 13 (IL13) to final concentrations of 20 ng/mL to differentiate M0 macrophages to alternatively-activated M2 macrophages. Culture the macrophages for 48 h in the incubator at 37 °C, 5% CO_2_.

It is recommended to follow the timeline presented in Table 1 if it is necessary to begin an experiment with different subtypes of macrophages simultaneously. The procedure of generating M0, M1 and M2 macrophages for an experiment with one starting point takes 5 days (Table 1).

**Table 1:** Timeline for generation of M0, M1 and M2 macrophages from THP-1 cells

Macrophage subtype	Day 0	Day 1	Day 2	Day 3	Day 4
**M0**	-	-	Seed 4 × 10^5^ THP-1 cells/well (6-well plate format). Incubate with 150 nM of PMA for 24 h.	Wash cells with PBS and add 2 mL of fresh culture medium (without PMA).	**Established M0**
**M1**	-	Seed 4 × 10^5^ THP-1 cells/well (6-well plate format). Incubate with 150 nM of PMA for 24 h.	Wash cells with PBS and add 2 mL of fresh culture medium (without PMA).	Add 100 ng/mL of LPS and 20 ng/mL of IFNgamma (final concentrations) for 18 h.	**Established M1**
**M2**	Seed 4 × 10^5^ THP-1 cells/well (6-well plate format). Incubate with 150 nM of PMA for 24 h.	Wash cells with PBS and add 2 mL of fresh culture medium (without PMA).	Add 20 ng/mL of IL4 and 20 ng/mL of IL13 (final concentrations) for 48 h.	-	**Established M2**

15. The successful generation of M0, M1 and M2 macrophages can be validated using RT-qPCR (see *Validation of macrophage generation (option 1)* section) and/or flow cytometry (see *Validation of macrophage generation (option 2)* section).

The representative pictures of M0, M1 and M2 macrophages are shown in Fig. 1B. For helpful tips on THP-1 culturing and macrophage generation, refer to *Notes 1* and *4*.

### Validation of Macrophage Generation (Option 1)

#### RT-qPCR

1. Wash cells with PBS.
2. Isolate RNA from the cells using the EurX GeneMATRIX Universal RNA Purification Kit according to the manufacturer’s protocol.
3. Measure RNA concentration and purity.
4. Perform cDNA synthesis using the High-Capacity cDNA Reverse Transcription Kit following the manufacturer’s protocol. Use 500 ng of RNA for reverse transcription reaction.
5. Prepare the RT-qPCR reaction by mixing 5µL of iTaq Universal SYBR Green Supermix (x2) BioRad, 0.5 µL of each forward and reverse primers (final concentration of primers: 500 nM), and 3 µL of nuclease-free H_2_O per 1 well (96-well PCR plate). Prepare samples in triplicates. Sequences of primers are presented in Table 2.
6. Add 1 µL of cDNA per 1 well (exclude negative control wells).
7. Spin down the plate with reaction mix and cDNA (1100 x g for 1 min).
8. Set the reaction conditions on the machine according to Table 3 and run RT-qPCR.
9. Analyze the results using the 2^-ΔΔ*CT*^ method (CT = threshold cycle).
10. Check if changes in relative gene expression levels for examined macrophage samples are significant compared to the controls (M0 macrophages). The results of RT-qPCR validation are shown in Fig. 1C.

**Table 2:** List of primers

Target mRNA	Forward primer	Reverse primer
TNFalpha	CTCTTCTGCCTGCTGCACTTTG	ATGGGCTACAGGCTTGTCACTC
CXCL10	GAAAGCAGTTAGCAAGGAAAGGTC	ATGTAGGGAAGTGATGGGAGAGG
CD206	CCATGGACAATGCGCGAGCG	CACCTGTGGCCCAAGACACGT
CD163	TTTGTCAACTTGAGTCCCTTCAC	TCCCGCTACACTTGTTTTCAC
GAPDH	CCATGGAGAAGGCTGGGG	GTCCACCACCCTGTTGCTGTA

**Table 3:** RT-qPCR reaction conditions

Step	Tempretaure [°C]	Time [s]	Number of cycles
Pre-incubation	95	5	1
Amplification	95	10	45
	60	10	
	72	10	
Melting curve	95	5	1
	65	60	
	97	5	
Cooling	40	30	1

### Validation of Macrophage Generation (Option 2)

#### Flow Cytometry

1. Wash cells with PBS and detach with trypsin.
2. Once cells are detached, add culture medium and transfer the cells to 15 mL tubes.
3. Centrifuge at 330 x g for 5 min.
4. Discard supernatants and resuspend cells in 3 mL of PBS.
5. Centrifuge at 330 x g for 5 min.
6. Discard supernatants and resuspend cells in 200 µL of PBS.
7. Transfer 200 µl of cell suspension to a 96-well U-bottom plate.
8. Centrifuge at 330 x g for 5 min.
9. Discard supernatants gently to avoid pellets detaching.
10. Resuspend cells in 50 µL/well FACS buffer.
11. Add 3 µL/well human TruStain FcX blocking solution to cells resuspended in FACS buffer, vortex gently.
12. Incubate for 15 min at room temperature.
13. Add 3 µL of each antibody (details in *Reagent, Solutions and Materials* section) to cells resuspended in FACS buffer (M1 marker: PE-CD38; M2 marker: BV421-CD206). Prepare the unstained control sample without the antibody.
14. Vortex gently.
15. Incubate for 20 min at 4 °C in the dark.
16. Centrifuge at 330 x g for 5 min.
17. Discard supernatants gently to avoid pellets detaching.
18. Resuspend cells in 200 µl/well FACS buffer.
19. Centrifuge at 330 x g for 5 min.
20. Discard supernatants gently to avoid pellets detaching.
21. Repeat 2 times steps 18-20.
22. Resuspend cells in 200 µl/well FACS Sheath Fluid.
23. Filter cell suspensions through FACS tubes with a strainer.
24. After filtering, divide samples into 3 technical replicates (separate FACS tubes).
25. Measure the samples within 0.5 h using the flow cytometer. The results of the flow cytometry analysis are shown in Fig. 1D and E.

**Fig. 2:**
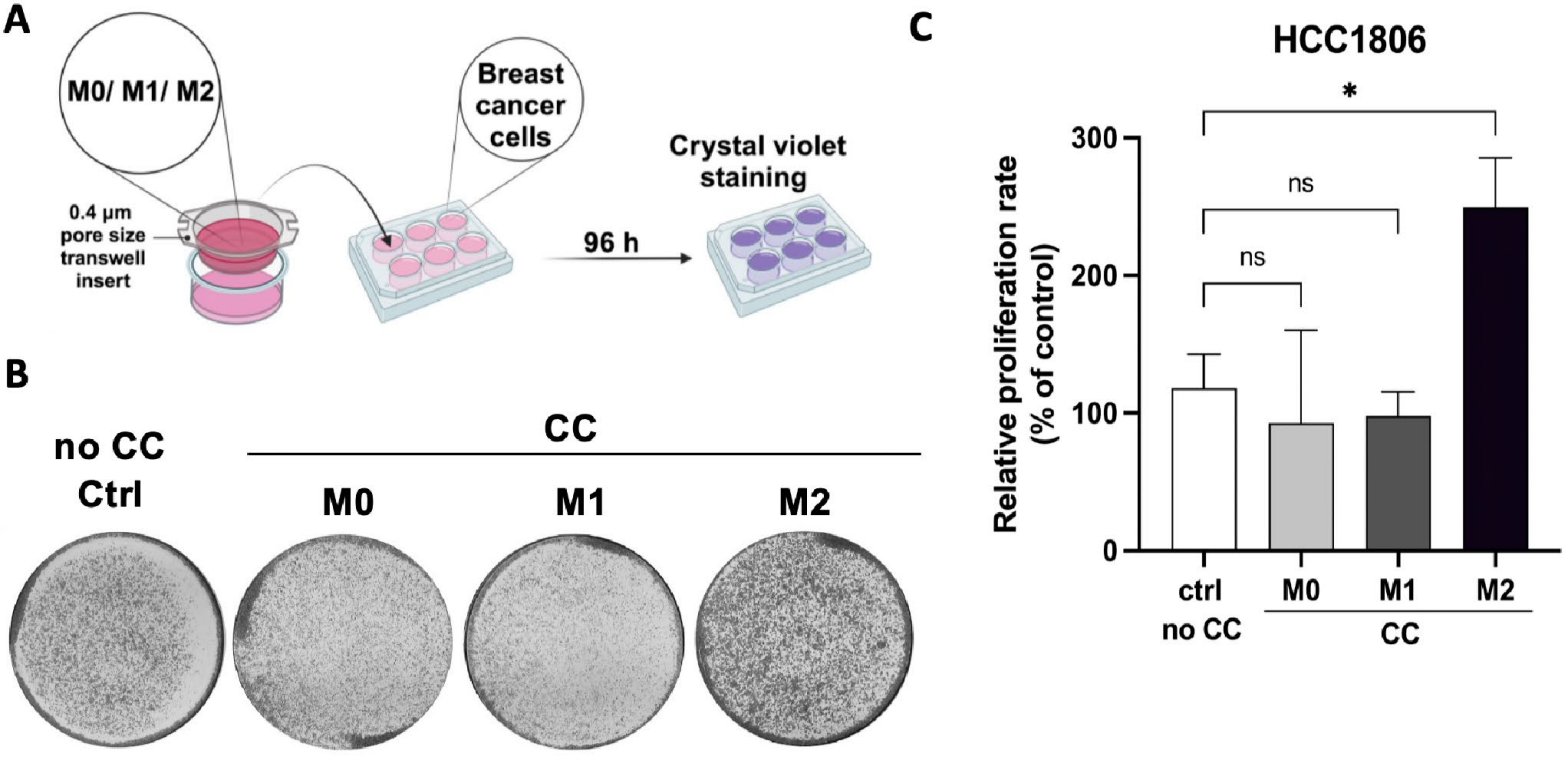
M2 macrophages increase the clonogenic potential of breast cancer cells. **A** Schematic illustration of the colony formation assay for breast cancer cells co-cultured with macrophages. **B, C** M0, M1, and M2 macrophages were generated in 0.4 μm pore size transwell inserts. HCC1806 cells were pre-seeded into 6-well plates. After attachment to the plate, HCC1806 were co-cultured (CC) with M0, M1 and M2 macrophages. Control conditions included HCC1806 cells grown without macrophages (Ctrl no CC). After 4 days, HCC1806 cells were stained with crystal violet (**B)**. The colony number was quantified by measuring the absorbance after the solubilization of the dye (OD540 nm) (**C)**. Data represent mean results from three independent experiments (error bars ± SD). Significance was calculated using one-way ANOVA followed by the Tukey multiple comparisons test, *p < 0.05

**Fig. 3:**
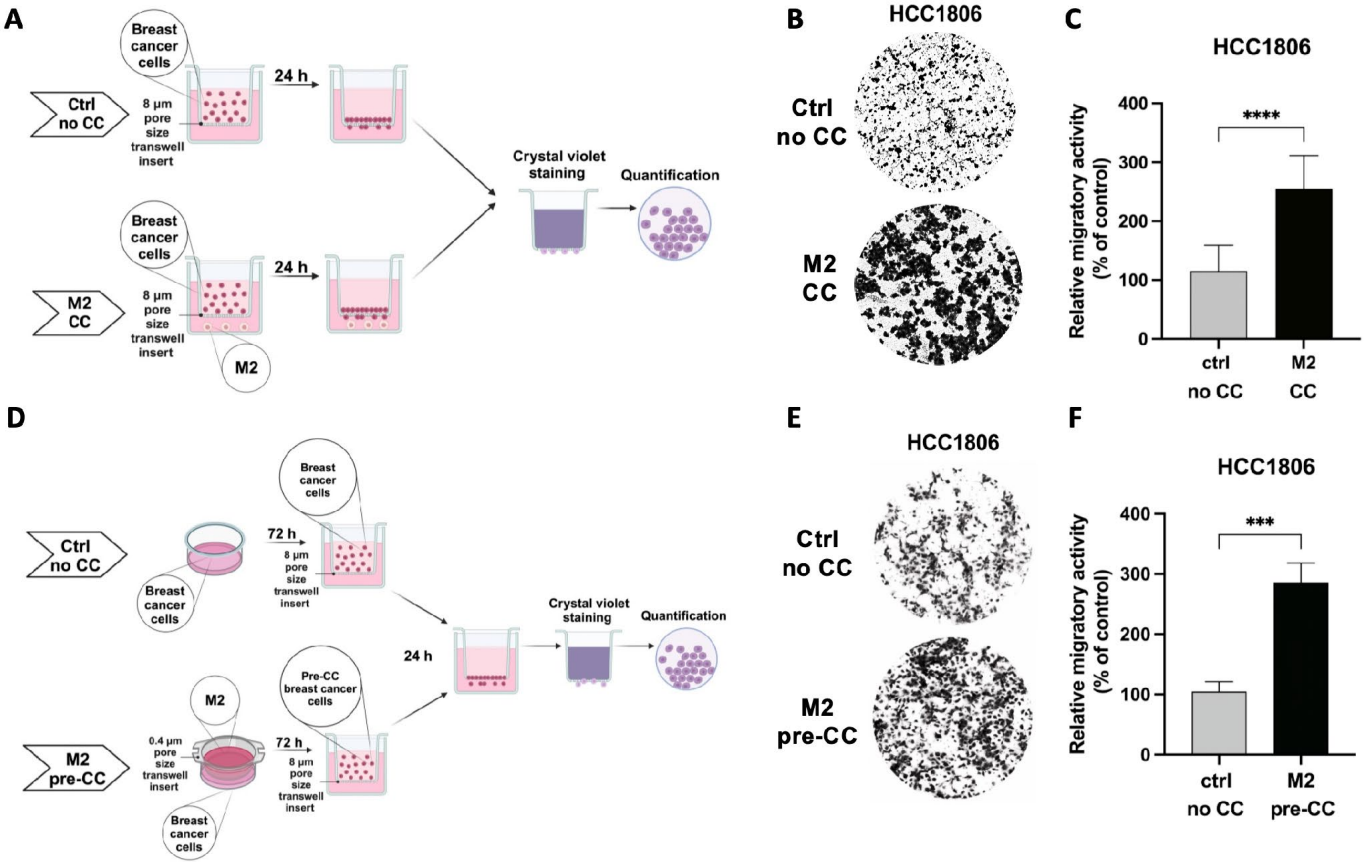
M2 macrophages increase the migratory potential of breast cancer cells. **A** Schematic illustration of the migration assay for breast cancer cells migrating directly to M2 macrophages. **B, C** M2 macrophages were generated in 12-well plates. HCC1806 cells were seeded into 8 μm pore size transwell inserts, and the inserts were placed into the wells with M2 macrophages. Control conditions included HCC1806 cells seeded into 8 μm pore size transwell inserts and placed into the wells without macrophages (Ctrl no CC). After 24 h, HCC1806 that migrated were stained with crystal violet and the pictures were taken (**B**). Results were quantified by ImageJ (**C**). Data represent mean results from three independent experiments (error bars ± SD). Significance was calculated using one-way ANOVA followed by the Tukey multiple comparisons test, ****p < 0.0001. **D** Schematic illustration of the migration assay for breast cancer cells after pre-co-culture with M2 macrophages. **E, F** M2 macrophages were generated in 0.4 μm pore size transwell inserts. HCC1806 cells were pre-seeded into 6-well plates. After attachment to the plate, HCC1806 were co-cultured with M2 macrophages. Control conditions included HCC1806 cells grown without macrophages (Ctrl no CC). After 3 days, HCC1806 cells were trypsinized and seeded into 8 μm pore size transwell inserts for migration assay. 24 h later, HCC1806 that migrated were stained with crystal violet, and the pictures were taken (**E**). Results were quantified by ImageJ (**F**). Data represent mean results from three independent experiments (error bars ± SD). Significance was calculated using one-way ANOVA followed by the Tukey multiple comparisons test, ***p < 0.001

THP-1-derived M0, M1 and M2 macrophages can be utilized in various phenotypic assays to determine the influence of macrophages on cancer cells’ behavior. Below, we provide detailed protocols for testing the clonogenic potential (Fig. 2A) and migratory activity (Fig. 3A and D) of breast cancer cells upon the co-culture with macrophages.

### Co-Culture-Based Colony Formation Assay with Breast Cancer Cells and Macrophages

1. **(Day 0–4)** Generate M0, M1 and M2 macrophages in 0.4 μm pore size transwell inserts following the *Generation of M0, M1 and M2 macrophages* section. Follow the timeline presented in Table 1.
2. **(Day 3)** Pre-seed HCC1806 cells into the transwell-suitable 6-well plate in a culture medium (see *Note 3*). Depending on the duration of the colony formation assay, seed the desired number of cells. For example, HCC1806 are seeded at the confluence of 5 × 10^4^ cells per well for the 96 h co-culture experiment. Seed 1 additional well with HCC1806 as a control (no co-culture conditions).
3. **(Day 4)** Wash pre-seeded HCC1806 twice with PBS and add 2 mL of fresh RPMI-1640 medium supplemented by 1% FBS to each well.
4. Wash 0.4 μm pore size transwell inserts with macrophages twice with PBS.
5. Place 0.4 μm pore size transwell inserts with macrophages into the wells with breast cancer cells. Handle insets at their edges using forceps in sterile conditions.
6. Add 2 mL of fresh RPMI-1640 medium supplemented by 1% FBS into each 0.4 μm pore size transwell insert.
7. Culture cells for 96 h at 37 °C, 5% CO_2_.
8. **(Day 8)** Aspirate off medium from inserts and wells using a glass Pasteur pipette.
9. Discard the 0.4 μm pore size transwell inserts with macrophages.
10. Wash wells with HCC1806 with PBS, add 2 mL of ice-cold methanol to each well, and incubate for 10 min.
11. Aspirate off methanol, add 2 mL of Formal-Fixx™ 1x solution to each well, and incubate for 10 min.
12. Aspirate off the Formal-Fixx™ 1x solution and stain with 0.5% crystal violet solution for 10 min.
13. Aspirate off crystal violet solution, wash wells with miliQ water and leave plates to dry overnight.
14. **(Day 9)** Take pictures (Fig. 2B) and quantify the results using ImageJ or resolving the crystals in 10% acetic acid, followed by absorbance measurements (OD540 nm) (Fig. 2C).

### Co-Culture-Based Migration Assays for Breast Cancer Cells

We propose two options for migration assay. The first option is a migration assay directly to M2 macrophages to investigate if the factors secreted by M2 macrophages can act as attractants for breast cancer cells (Fig. 3A). The second option involves co-culturing breast cancer cells with M2 macrophages, followed by the migration assay (Fig. 3D). It can be used to determine whether the phenotypic changes that occur in breast cancer cells during the co-culture with M2 macrophages are long-lasting and detectable even without the presence of M2 macrophages. Both methods yield similar results.

### Migration Assay for Breast Cancer Cells Migrating Directly to M2 Macrophages

1. **(Day 0–4)** Generate M2 macrophages in the cell culture plates following the *Generation of M0, M1 and M2 macrophages* section. For 12-well format plates, seed 2 × 10^5^ THP-1 cells/well in 1mL of culture medium.
2. **(Day 4)** Aspirate off medium from wells with M2 macrophages using a glass Pasteur pipette.
3. Add 1 mL of fresh RPMI-1640 medium supplemented with 1% FBS to wells with M2 macrophages.
4. Place 8 μm pore size transwell inserts (12-well format) into the wells with M2 macrophages (see *Note 3*). Include well without M2 macrophages as control (no co-culture conditions).
5. Seed 1.5 × 10^5^ HCC1806 cells per 8 μm pore size transwell insert in 1 mL of fresh RPMI-1460 medium without FBS.
6. Culture cells for 24 h at 37 °C, 5% CO_2_.
7. **(Day 5)** Aspirate off medium from the 8 μm pore size transwell inserts using a glass Pasteur pipette.
8. Transfer the inserts to a new 12-well plate with ice-cold methanol and incubate for 10 min.
9. Aspirate off methanol and incubate the inserts with Formal-Fixx™ 1x solution for 10 min.
10. Aspirate off Formal-Fixx™ 1x solution and stain the inserts with 0.5% crystal violet solution for 10 min.
11. Aspirate off crystal violet solution and wash the inserts with miliQ water.
12. Clean the insides of the inserts using earbuds to remove cells that didn’t migrate.
13. Leave the inserts to dry overnight.
14. **(Day 6)** Take pictures of the 8 μm pore size transwell inserts (Fig. 3B) and quantify the results by ImageJ (Fig. 3C).

### Migration Assays Performed on Breast Cancer Cells after the Co-Culture with M2 Macrophages

1. **(Day 0–4)** Generate M2 macrophages in 0.4 μm pore size transwell inserts following the *Generation of M0, M1 and M2 macrophages* Sect. 
2. **(Day 3)** Pre-seed HCC1806 into a transwell-suitable 6-well plate in culture medium (see *Note 3*). For 72 h co-culture, HCC1806 are seeded at the confluence of 1.5 × 10^5^ cells per well. Include 1 additional well with HCC1806 as a control (no co-culture condition).
3. **(Day 4)** Aspirate off medium from 0.4 μm pore size transwell inserts with M2 macrophages and from 6-well plate with pre-seeded HCC1806 using glass Pasteur pipettes.
4. Wash M2 macrophages and HCC1806 twice with PBS.
5. Add 2 mL of fresh RPMI*-*1460 medium supplemented with 1% FBS to the 6-well plates with HCC1806.
6. Place 0.4 μm pore size transwell inserts with M2 macrophages into the 6-well plate with HCC1806. Handle insets at their edges using forceps in sterile conditions.
7. Add 2 mL of fresh RPMI*-*1460 medium supplemented with 1% FBS to 0.4 μm pore size transwell inserts with M2 macrophages.
8. Culture cells for 72 h at 37 °C, 5% CO_2_.
9. **(Day 7)** Aspirate off medium from inserts and wells using a glass Pasteur pipette.
10. Discard the 0.4 μm pore size transwell inserts with M2 macrophages.
11. Wash the wells with HCC1806 cells with PBS, add 0.5 mL of trypsin per well and incubate at 37 °C, 5% CO_2_ for 5-10 min.
12. Add 2 mL of fresh culture medium to each well to wash out the trypsin and transfer HCC1806 cell suspensions to 15 mL tubes.
13. Centrifuge tubes at 330 x g for 5 min.
14. Aspirate off supernatants very carefully using a glass Pasteur pipette.
15. Resuspend cell pellets in 0.5 mL of fresh RPMI-1460 medium without FBS and count cells using trypan blue.
16. Prepare a 12-well plate for the migration assay by adding 1 mL of fresh RPMI-1640 medium supplemented by 20% FBS to wells and inserting 8 μm pore size transwell inserts (12-well format, see *Note 3*).
17. Seed 1.5 × 10^5^ HCC1806 cells into 8 μm pore size transwell inserts in 1 mL of RPMI-1640 medium without FBS.
18. Culture cells for 24 h at 37 °C, 5% CO_2_.
19. **(Day 8)** Aspirate off medium from the 8 μm pore size transwell inserts using a glass Pasteur pipette.
20. Transfer the inserts to a new 12-well plate with ice-cold methanol and incubate for 10 min.
21. Aspirate off methanol and incubate the inserts with Formal-Fixx™ 1x solution for 10 min.
22. Aspirate off Formal-Fixx™ 1x solution and stain the inserts with 0.5% crystal violet solution for 10 min.
23. Aspirate off crystal violet solution and wash the inserts with miliQ water.
24. Clean the insides of the inserts using earbuds to remove cells that didn’t migrate.
25. Leave the inserts to dry overnight.
26. **(Day 9)** Take pictures of the 8 μm pore size transwell inserts (Fig. 3E) and quantify the results by ImageJ (Fig. 3F).

## Notes

1. THP-1 is a monocytic suspension cell line that can form clusters. To avoid this issue, it is important to process sensitive cells, like THP-1, immediately after thawing to minimize contact with the freezing medium, which contains DMSO. The freezing medium should be washed out as quickly as possible using a culture medium. Centrifuge THP-1 at a maximum of 190 x g for 5 min. THP-1 are grown in non-treated culture flasks.
2. PMA is photosensitive – protect from light.
3. Based on our experience, the quality of the transwell inserts used for phenotypic assays (co-culture and migration) is crucial. Always use plates recommended by the inserts’ manufacturer to prevent direct contact between the inserts and the plate. Both membrane types of 0.4 μm pore size transwell inserts, polycarbonate and PET, work well and yield similar results.
4. To achieve consistency in the macrophage generation process, it is important to consider several factors. A recommended practice is to culture THP-1 cells for no more than 30 passages to minimize variability in macrophage phenotype. Moreover, the culture medium should be changed 2–3 times per week, and the optimal density for culturing THP-1 is 5 × 10^5^ to 1 × 10^6^ cells/mL. To obtain repetitive data, it is crucial to regularly check the phenotype (expression of markers) of macrophages used in the phenotypic assays.

## Discussion

We have developed protocols to perform phenotypic assays using the transwell co-culture method for breast cancer cells and THP-1-derived macrophages. These methods enable us to effectively investigate the impact of macrophages on the clonogenic potential and migratory activity of breast cancer cells. The co-culture protocols can also be modified for various assays, such as the invasion assay, wound healing assay, or drug response. The use of transwell inserts in co-culture enables the isolation of protein or RNA from both cell types, allowing for immunoblotting or gene expression analysis. We used the HCC1806 breast cancer cell line here, but these protocols can be adapted for other adherent cancer cell lines.

The results obtained by us verified the utility of the proposed protocols. We confirmed the successful generation of the M1 and M2 phenotypes by two independent techniques (Fig. 1C-E). We also showed that THP-1 could be a useful model for studying the interactions between cancer cells and macrophages. Our study demonstrated that THP-1-derived M2 macrophages increase the clonogenic potential and migratory activity of breast cancer cells, which aligns with previous studies using THP-1 as well as other macrophage models (Figs. 2C and 3C and F) [9, 12, 19, 20]. In summary, the optimized co-culture protocols for cancer cells and macrophages will help study the cross-talk between tumors and their microenvironment.

## Abbreviations

FBS: fetal bovine serum
IFNgamma: interferon gamma
IL4: interleukin 4
IL13: interleukin 13
LPS: lipopolysaccharide
PBS: phosphate-buffered saline
PMA: phorbol 12-myristate 13-acetate
TAMs: tumor-associated macrophages
TME: tumor microenvironment
TNFalpha: tumor necrosis factor alpha
